# Surveillance of tick-borne bacteria infection in ticks and forestry populations in Inner Mongolia, China

**DOI:** 10.3389/fpubh.2024.1302133

**Published:** 2024-02-29

**Authors:** Like Duan, Lin Zhang, Xuexia Hou, Zihao Bao, Yu Zeng, Lijuan He, Zeliang Liu, Haijian Zhou, Qin Hao, Aiying Dong

**Affiliations:** ^1^National Institute for Communicable Disease Control and Prevention, Chinese Center for Disease Control and Prevention, National Key Laboratory of Intelligent Tracking and Forecasting for Infectious Diseases, Beijing, China; ^2^Affiliated Hospital of North China University of Science and Technology, Tangshan, China

**Keywords:** tick-borne bacteria, *Ixodes persulcatus*, co-infection, spotted fever group Rickettsia, Inner Mongolia

## Abstract

Ticks are one of the most important vectors that can transmit pathogens to animals and human beings. This study investigated the dominant tick-borne bacteria carried by ticks and tick-borne infections in forestry populations in Arxan, Inner Mongolia, China. Ticks were collected by flagging from May 2020 to May 2021, and blood samples were collected from individuals at high risk of acquiring tick-borne diseases from March 2022 to August 2023. The pooled DNA samples of ticks were analyzed to reveal the presence of tick-borne bacteria using high-throughput sequencing of the 16S rDNA V3–V4 region, and species-specific polymerase chain reaction (PCR) related to sequencing was performed to confirm the presence of pathogenic bacteria in individual ticks and human blood samples. All sera samples were examined for anti-SFGR using ELISA and anti-*B. burgdorferi* using IFA and WB. A total of 295 ticks (282 *Ixodes persulcatus* and 13 *Dermacentor silvarum*) and 245 human blood samples were collected. *Rickettsia*, *Anaplasma*, *Borrelia miyamotoi*, and *Coxiella endosymbiont* were identified in *I. persulcatus* by high-throughput sequencing, while *Candidatus R. tarasevichiae* (89.00%, 89/100), *B. garinii* (17.00%, 17/100), *B. afzelii* (7.00%, 7/100), and *B. miyamotoi* (7.00%, 7/100) were detected in *I. persulcatus*, as well the dual co-infection with *Candidatus R. tarasevichiae* and *B. garinii* were detected in 13.00% (13/100) of *I. persulcatus*. Of the 245 individuals, *B. garinii* (4.90%, 12/245), *R. slovaca* (0.82%, 2/245), and *C. burnetii* (0.41%, 1/245) were detected by PCR, and the sequences of the target genes of *B. garinii* detected in humans were identical to those detected in *I. persulcatus*. The seroprevalence of anti-SFGR and anti-*B. burgdorferi* was 5.71% and 13.47%, respectively. This study demonstrated that *Candidatus R. tarasevichiae* and *B. garinii* were the dominant tick-borne bacteria in *I. persulcatus* from Arxan, and that dual co-infection with *Candidatus R. tarasevichiae* and *B. garinii* was frequent. This is the first time that *B. miyamotoi* has been identified in ticks from Arxan and *R. solvaca* has been detected in humans from Inner Mongolia. More importantly, this study demonstrated the transmission of *B. garinii* from ticks to humans in Arxan, suggesting that long-term monitoring of tick-borne pathogens in ticks and humans is important for the prevention and control of tick-borne diseases.

## Introduction

Ticks are obligate hematophagous ectoparasites of terrestrial vertebrates that can transmit various pathogens to humans and are considered to be the second most notable vectors worldwide after mosquitoes (1). By the end of 2018, 103 tick-borne pathogens were detected in China, of which 65 were newly identified in the past two decades (2). Notable bacteria such as *Rickettsia*, *Borrelia*, *Coxiella*, *Anaplasma*, and *Ehrlichia* (2–4), have been detected in a wide range of tick species that can cause spread of relevant zoonotic diseases. Annually expanding geographic ranges and increasing populations of ticks, partly driven by climate change, demographic growth, and increased overseas travel and trade, have become serious threats to public health (5, 6). However, systematic and comprehensive investigations of tick-borne pathogens have not been conducted in tick habitats in China because of the limitations of investigation regions and research technology.

Inner Mongolia Autonomous Region, located in northern China, is well-known for its various landscapes and is one of the natural foci of various tick-borne diseases in China (7–10). Arxan, on the border between China and Mongolia in the northeastern Inner Mongolia, features abundant wildlife resources and a developed livestock industry that provides a suitable environment for ticks to survive and reproduce. Trade activities at border crossings and tourism have partly increased the risk of tick-borne diseases. For instance, the average annual incidence of tick-borne encephalitis (TBE) in Arxan from 2006 to 2013 was 188.48/1000000, indicating that Arxan has the highest incidence of TBE in China (11). However, tick-borne bacteria in Arxan have not been studied in recent years. Therefore, investigating the status of bacterial infections in ticks and humans plays an important role in the prevention and control of tick-borne diseases in Arxan.

In this study, we performed high-throughput sequencing of the 16S rDNA V3–V4 region to detect tick-borne bacteria carried by ticks, and species-specific polymerase chain reaction (PCR) was performed to confirm the presence of pathogenic bacteria in ticks and participants at a high risk of tick-borne diseases in Arxan. Furthermore, antibodies against dominant tick-borne bacteria were detected using Enzyme-Linked Immunosorbent Assay (ELISA), Indirect Immunofluorescence Assay (IFA), and Western Blotting (WB) assays, providing complete and comprehensive monitoring of tick-borne bacteria and a theoretical basis for the prevention and control of tick-borne diseases.

## Materials and methods

### Collection and identification of ticks

**Figure 1:**
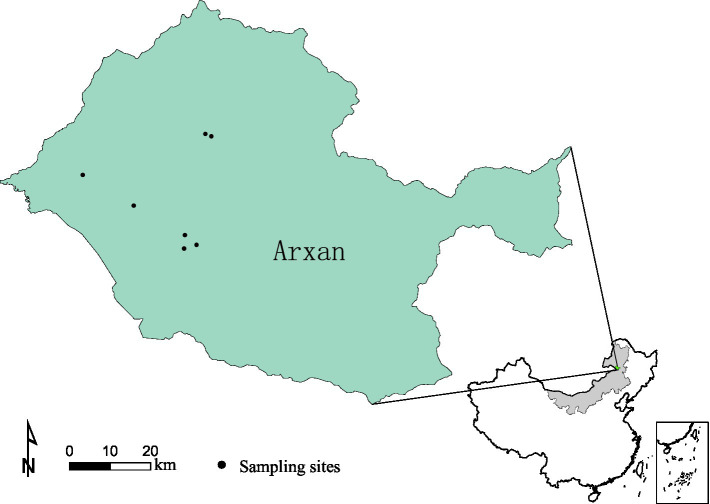
Map of the tick sampling sites in Arxan, Inner Mongolia, China.

Free-living ticks were collected from ground vegetation by dragging white cloth in May 2020 and 2021 in Arxan, Inner Mongolia, China (Figure 1). All collected ticks were placed in breathable and moistened bottles and transported to the laboratory as soon as possible at room temperature (20–25°C). Tick species were identified based on morphological characterization and verified using sequences of mitochondrial 16S rDNA genes, as previously described (12). After identification, the ticks were stored at −80°C until use.

### Collection of human blood samples

Patients with tick-borne diseases mostly work in the field, including as forestry workers (13–15). These workers had a high possibility of tick exposure and therefore, were defined as groups at high risk of acquiring tick-borne diseases. We simultaneously collected anticoagulated (EDTA blood) and non-anticoagulant blood samples from 245 participants (232 males and 13 females) working in the field from March 2022 to August 2023. The workplaces of these participants were Tianchi forest farm (8.57%, 21/245), Sandur forest farm (15.92%, 39/245), Lixin forest farm (16.33%, 40/245), Irsh forest farm (28.16%, 69/245), Xing’an Service Area (8.98%, 22/245), Jinjianggou Service Area (11.84%, 29/245) and Transportation Company (10.20%, 25/245), respectively. The median age of all participants was 53.00 years (range 23–60 years). All the participants provided written informed consent to participate in this study. Sera were isolated from non-anticoagulant blood samples and stored at −20°C for serological tests. Anticoagulated blood samples were used for DNA extraction.

### DNA extraction

All ticks were washed with 75% ethanol and rinsed thrice with sterile water to remove environmental contaminants. Except for the 182 *I. persulcatus* samples used for the isolation and culture of *B. burgdorferi*, the remaining 100 *I. persulcatus* and 13 *D. silvarum* samples were used for DNA extraction. Individual tick samples were homogenized in 180 μL buffer ATL, then genomic DNA was extracted using the DNeasy Blood & Tissue Kit (QIAGEN, Germany) according to the manufacturer’s instructions. Similarly, the genomic DNA of human blood samples was extracted from anticoagulated blood using the DNeasy Blood & Tissue Kit (QIAGEN, Germany). Each time DNA extraction was performed, an extraction control (water) was added. All DNA samples from both ticks and human blood were stored at −80°C until use.

### High-throughput sequencing

Individual DNA samples of ticks were mixed in an equal volume (10 μL) to prepare pooled DNA samples (3 or 4 DNA samples for a pool). One hundred *I. persulcatus* DNA samples were mixed to prepare 32 pools (N1–N24 and N29–N36), and 13 *D. silvarum* DNA samples were mixed for 4 pools (N25–N28). All pooled DNA samples were sent to Biomarker Technologies Co., Ltd. (Beijing, China) for high-throughput sequencing of the 16S rDNA V3–V4 region on an Illumina NovaSeq 6,000 platform (Illumina, San Diego, CA, United States) with a sequencing depth of 80,000. The bioinformatics analysis of raw data was performed with the aid of the BMK Cloud (Biomarker Technologies Co., Ltd., Beijing, China). Raw data were filtered according to the quality of a single nucleotide using Trimmomatic (version 0.33) (16). Primer sequences were identified and removed using Cutadapt (version 1.9.1) (17). Clean reads obtained from the previous steps were assembled using USEARCH (version 10) (18) and followed by chimera removal using UCHIME (version 8.1) (19). Effective reads were generated and used for subsequent analyses. Sequences with similarity ≥97% were clustered into the same operational taxonomic unit (OTU) by USEARCH (version 10). Taxonomic annotation of the OTUs was performed based on the Naive Bayes classifier in QIIME2 (20) using the SILVA database (release 132) (19), following which a relative abundance table and histogram for each sample at the level of the phylum, class, order, family, genus, and species were calculated and displayed. Alpha diversity, including Chao1, ACE, Shannon, and Simpson indices, were calculated and displayed using QIIME2 and R software.

### Polymerase chain reaction

According to the results of high-throughput sequencing and tick-borne bacteria prevalently detected in Inner Mongolia, the presence of *Borrelia burgdorferi*, *B. miyamotoi*, *Anaplasma phagocytophilum*, spotted fever group rickettsia, *Ehrlichia chaffeensis*, and *Coxiella burnetii* were tested in ticks and human blood samples using nested PCR or traditional PCR. The PCR primer sequences are listed in Table 1. PCR was performed using a PCR system (SensoQuest, Germany). The PCR system was totally 25 μL, including 12.5 μL Premix Taq^™^ (TaKaRa), 1 μL upper primer (10 μm), 1 μL lower primer (10 μm), 5 μL each individual DNA sample and 5.5 μL ddH_2_O. Positive (recombinant plasmid containing the target gene), negative (water), and extraction controls were used for PCR amplification in each PCR experiment. For nested PCR, 1 μL of the primary PCR production was used as the template for the second round. All amplified products were electrophoresed on a 1.5% agarose gel and the positive products were purified and sequenced. The acquired nucleotide sequences were submitted to National Center for Biotechnology Information Genbank and compared with available sequences deposited using the Basic Local Assignment Search Tool.^1^ Additionally, human blood samples that were positive for SFGR based on nested PCR were tested using qPCR targeting *gltA* gene. qPCR was performed using a Probe qPCR mix (Premix Ex Taq^™^,TaKaRa) on a LightCycler 480 System (Roche Diagnostics, United States).

**Table 1:** Target genes and primer sequences used for PCR.

Bacteria	Target gene	Primer name	Sequence (5’–3’)	Size (bp)	References
B.burgdorferi	5S-23S rRNA IGS	F1 R1 F2 R2	CGACCTTCTTCGCCTTAAAGC TAAGCTGACTAATACTAATTACCC TCCTAGGCATTCACCATA GAGTTCGCGGGAGA	255	(21)
B.miyamotoi	glpQ	F1 R1 F2 R2	CACCATTGATCATAGCTCACAG CTGTTGGTGCTTCATTCCAGTC GCTAGTGGGTATCTTCCAGAAC CTTGTTGTTTATGCCAGAAGGGT	424	(22)
SFGR	ompA	Rr190.70 Rr190.701 Rr190.70 Rr190.602	ATGGCGAATATTTCTCCAAAA GTTCCGTTAATGGCAGCATCT ATGGCGAATATTTCTCCAAAA AGTGCAGCATTGGCTCCCCCT	533	(23)
SFGR	gltA	Cs2d CsendR Rpcs877 Rpcs1258	ATGACCAATGAAAATAATAAT CTTATACTCTCTATGTACA GGGGACCTGCTCACGGCGG ATTGCAAAAAGTACAGTGAACA	341	(8)
SFGR	gltA	F R P	GTGAATGAAAGATTACACTATTTAT GTATCTTAGCAATCATTCTAATAGC 6-FAM-CTATTATGCTTGCGGCTGTCGGTTC-TAMRA	166	(24)
AP	16S rRNA	AP-F AP-R	GTCGAACGGATTATTCTTTATAGCTTG TATAGGTACCGTCATTATCTTCCCTAC	389	(25)
EC	16S rRNA	ECC ECB H3 H1	AGAACGAACGCTGGCGGCAAGCC CGTATTACCGCGGCTGCTGGCA TATAGGTACCGTCATTATCTTCCCTAT CAATTGCTTATAACCTTTTGGTTATAAAT	389	(26, 27)
CB	IS1111	F1 R1 F2 R2	TACTGGGTGTTGATATTGC CCGTTTCATCCGCGGTG GTAAAGTGATCTACACGA TTAACAGCGCTTGAACGT	297	(28)

SFGR, spotted fever group rickettsia; AP, *Anaplasma phagocytophilum*; EC, *Ehrlichia chaffeensis*; CB, *Coxiella burnetii*.

### Phylogenetic analysis

Reference sequences of target genes downloaded from GenBank were aligned with the positive amplicon sequences using the ClustalW method with default parameters in MEGA11.0. One of the same nucleotide sequences detected in a tick or blood sample was selected as the representative sequence. Phylogenetic analysis was performed using the neighbor-joining algorithm, and bootstrap values were set for 1,000 replicates.

### Serological tests

Based on the results of the high-throughput sequencing and PCR assays, SFGR and *B. burgdorferi* were the dominant tick-borne bacteria in Arxan. All serum samples were tested anti-SFGR by ELISA and anti-*B. burgdorferi* using IFA and WB. ELISA kits acquired from Enzyme Immune Industrial Co., Ltd. (Jiangsu, China) were used to detect anti-SFGR IgG and IgM according to the manufacturer’s instructions. The cutoff value was the mean optical density (OD) of the negative control wells plus 0.15. Samples with OD values less than the cut-off value were considered negative. Samples with OD values greater than or equal to the cut-off value were considered positive.

IFA and WB were performed as previously described (29). First, all the serum samples were tested for anti-*B. burgdorferi* by IFA. A titer of ≥1/128 for IgG or 1:64 for IgM was considered positive. For IgG, samples that were positive at a titer of 1/64 but presented a weak fluorescence signal at a titer of 1/128 were considered equivocal. For IgM, samples that were positive at a titer of 1/32 but presented a weak fluorescence signal at a titer of 1/64 were considered equivocal. Subsequently, all sera with positive and equivocal (IgG or IgM) results were further analyzed by WB (both IgG and IgM). The positive criteria were at least one band of P83/100, P66, P58, P39, P30, OspC, OspA, and P17 in the IgG test and at least one band of P83/100, P58, P41, OspA, P30, OspC, and P17 in the IgM test (30).

### Statistical analysis

Statistical analysis was performed using Pearson’s *χ*^2^ test and *p* < 0.05 was considered significant.

## Results

### Identification of ticks

A total of 295 adult hard ticks were identified as *Ixodes persulcatus* (*n* = 282) and *Dermacentor silvarum* (*n* = 13) based on morphological characteristics and mitochondrial 16S rDNA sequences. The nucleotide sequence data of mitochondrial 16S rDNA in this study will appear in GenBank under the following accession numbers: OR841358–OR841362 for *I. persulcatus*, OR841363–OR841367 for *D. silvarum*.

### The overview of high-throughput sequencing

A total of 2,413,890 effective reads (range 69,960–76,736 reads, average 75434.06 reads) of high quality (Q20 > 98.94%, shown in Additional file 1: Supplementary Table S1) were obtained from 32 pooled *I. persulcatus* samples. Subsequently, the effective reads were clustered into 1,445 Operational Taxonomic Units (OTU), which belonged to 32 phyla, 79 classes, 191 orders, 348 families, and 594 genera. Moreover, 303,375 effective reads were acquired from four pooled DNA samples of *D. silvarum* (range 75,414–76,021 reads, average 75843.75 reads), all of which were of high quality (Q20 > 98.93%, shown in Additional file 1: Supplementary Table S1), and 1,430 OTUs were generated, belonging to 31 phyla, 78 classes, 190 orders, 346 families, and 588 genera.

**Figure 2:**
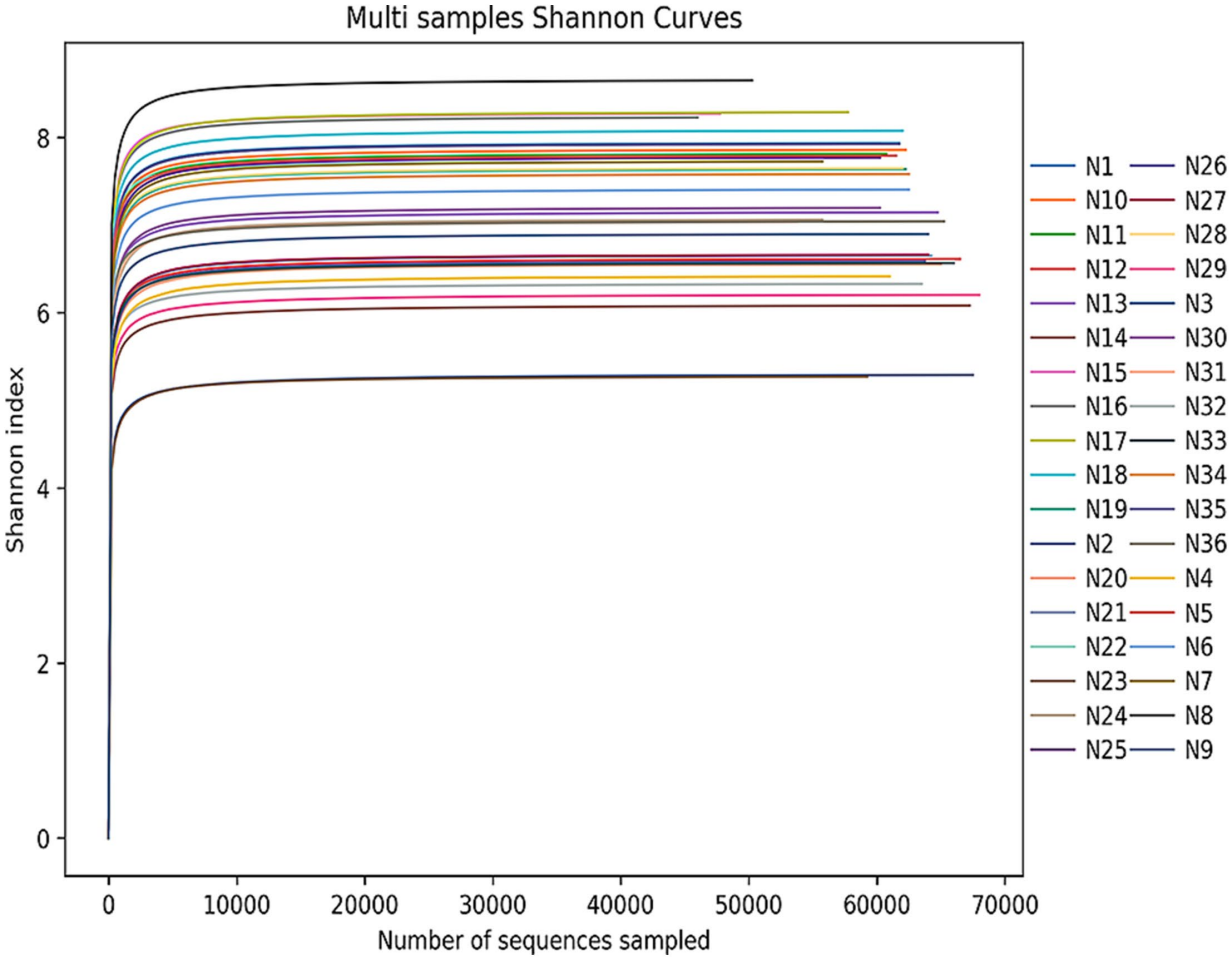
Shannon index rarefaction curve of 36 pools of *I. persulcatus* and *D. silvarum*. When the curve tends to be flat, it means that the sequencing data are sufficient for analysis, and the Shannon index will not increase with increasing sequencing data.

**Figure 3:**
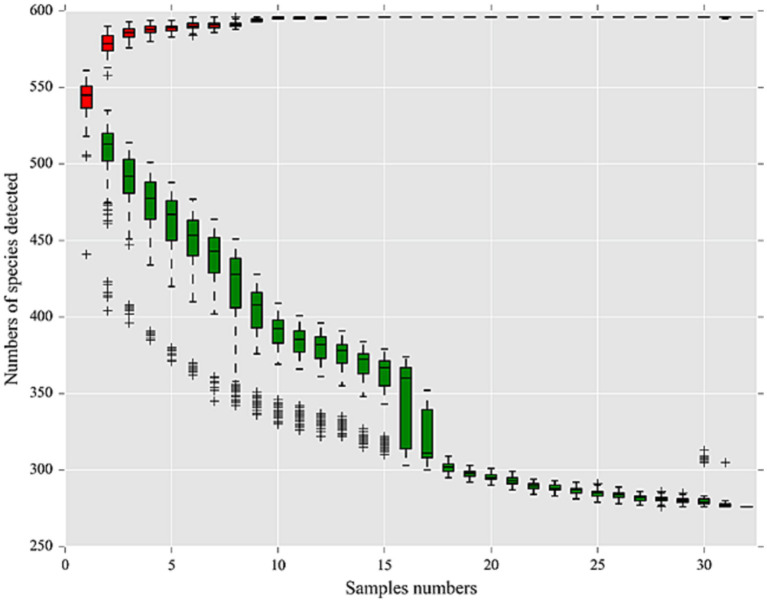
Species accumulation curve at genus level of 32 pools of *I. persulcatus*. Red boxes represent the accumulation curve of detected species numbers, and green boxes form the common curve of detected species numbers. When the curve tends to be flat, it means that the sample size is sufficient for the analysis.

Alpha diversity reflects species richness and diversity of individual samples. The Ace and Chao1 indices, which were calculated to measure species richness, were more than 1068.34 and 1098.66, respectively, in each pool (Additional file 2: Supplementary Table S2). Shannon and Simpson indices were used to measure species diversity, which were affected by species abundance and community evenness in the sample community. With the same species abundance, the greater the uniformity of each species in the community, the larger the Shannon and Simpson indices values, indicating higher species diversity in the sample. Shannon diversity rarefaction (Figure 2) reflects the microbial diversity of each sample at different sequencing amounts, and was used to determine whether the sequencing depth was sufficient. All pools were highly diverse, with Simpson indices more than 0.85 except for N23. The species accumulation curve reflects the relationship between the sample size and the number of annotated species, and can be used to determine whether the sample size was sufficient. The species accumulation curve of *I. persulcatus* at the genus level is shown in Figure 3. The number of *D. silvarum* pooled DNA samples was insufficient for the analysis of the species cumulative curve.

### Species annotation and taxonomic analysis

**Figure 4:**
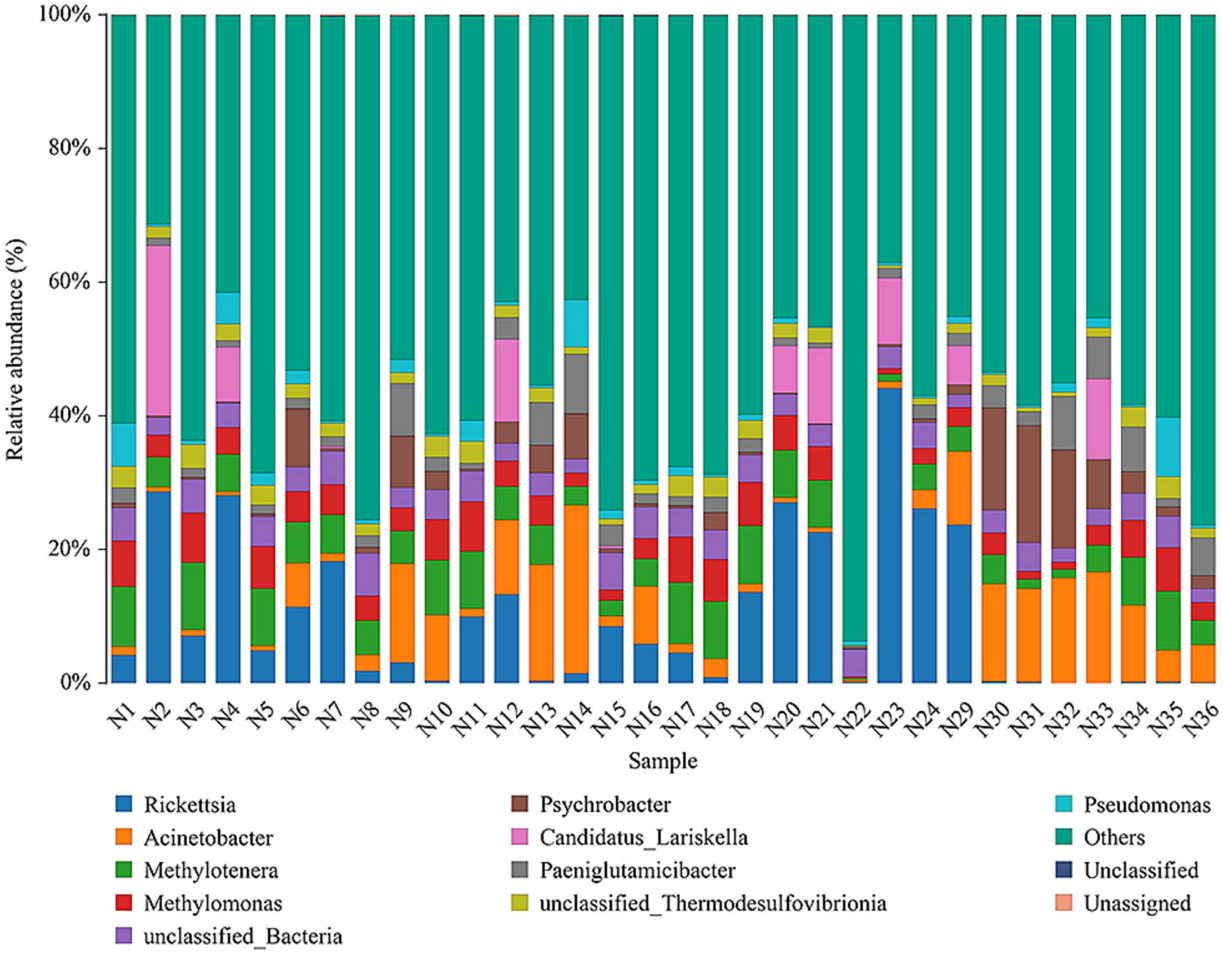
Relative abundances of top 10 microorganisms at the genus level in pooled *I. persulcatus* DNA samples.

Taxonomic assignment revealed that the relative abundances of the genera were highly variable in the microbiome of *I. persulcatus* (Figure 4). At least 594 genera were presented in *I. persulcatus*, including *Rickettsia*, *Acinetobacter*, *Methylotenera*, *Methylomonas*, *Psychrobacter*. *Rickettsia* was the most common genus, with a relative abundance >22.61% in the seven pools (Additional file 3: Supplementary Table S3). *Borrelia*, with a relative abundance of 0.15–0.35%, was detected in three pools and further identified as *B. miyamotoi*. Likewise, *Coxiella* (0.01–0.02%) was identified in five pools and further confirmed to be *Coxiella endosymbiont*. *Anaplasma* (0.01%) was present in two pools, but species-level annotation was lacking (Additional file 4, Supplementary Table S4).

Similarly, *Rickettsia* was the most abundant genus in *D. silvarum* (Additional file 3: Supplementary Table S3). *Coxiella* was identified in each *D. silvarum* pool and confirmed to be *Coxiella endosymbiont* at the species level (Additional file 4: Supplementary Table S4), whereas *Borrelia* was not detected in *D. silvarum*.

### Prevalence of tick-borne bacteria in ticks and humans

*Candidatus R. tarasevichiae* (89.00%, 89/100), *B. garinii* (17.00%, 17/100), *B. afzelii* (7.00%, 7/100), and *B. miyamotoi* (7.00%, 7/100) were detected in *I. persulcatus*, while *R. raoultii* (69.23%, 9/13) was detected in *D. silvarum* (Table 2). More importantly, *B. garinii* (4.90%, 12/245), *R. slovaca* (0.82%, 2/245), and *C. burnetii* (0.41%, 1/245) were detected in the human blood samples. Neither *Ehrlichia* DNA nor *Anaplasma* DNA was detected in ticks or human blood samples. The qPCR results showed a cycle threshold value of 38.86 for one of those 2 individuals that were detected positive for *R. slovaca* using nested PCR targeting *ompA* gene.

**Table 2:** Prevalence of tick-borne bacteria in ticks and humans.

Bacteria	Target gene	*I. persulcatus* (n = 100)	*D. silvarum* (n = 13)	Humans (*n* = 245)
Candidatus R. tarasevichiae	ompA	72 (72.00%)	–	–
	gltA	89 (89.00%)	–	–
	Total	89 (89.00%)	–	–
*R. raoultii*	ompA	–	9 (69.23%)	–
	gltA	–	8 (61.54%)	–
	Total	–	9 (69.23%)	–
*R. slovaca*	ompA	–	–	2 (0.82%)
	gltA	–	–	–
	Total	–	–	2 (0.82%)
*B. garinii*	5S-23S rRNA IGS	17 (17.00%)	–	12 (4.90%)
*B. afzelii*	5S-23S rRNA IGS	7 (7.00%)	–	–
*B. miyamotoi*	glpQ	7 (7.00%)	–	–
burnetii	IS1111	–	–	1 (0.41%)

### Phylogenetic analysis

**Figure 5:**
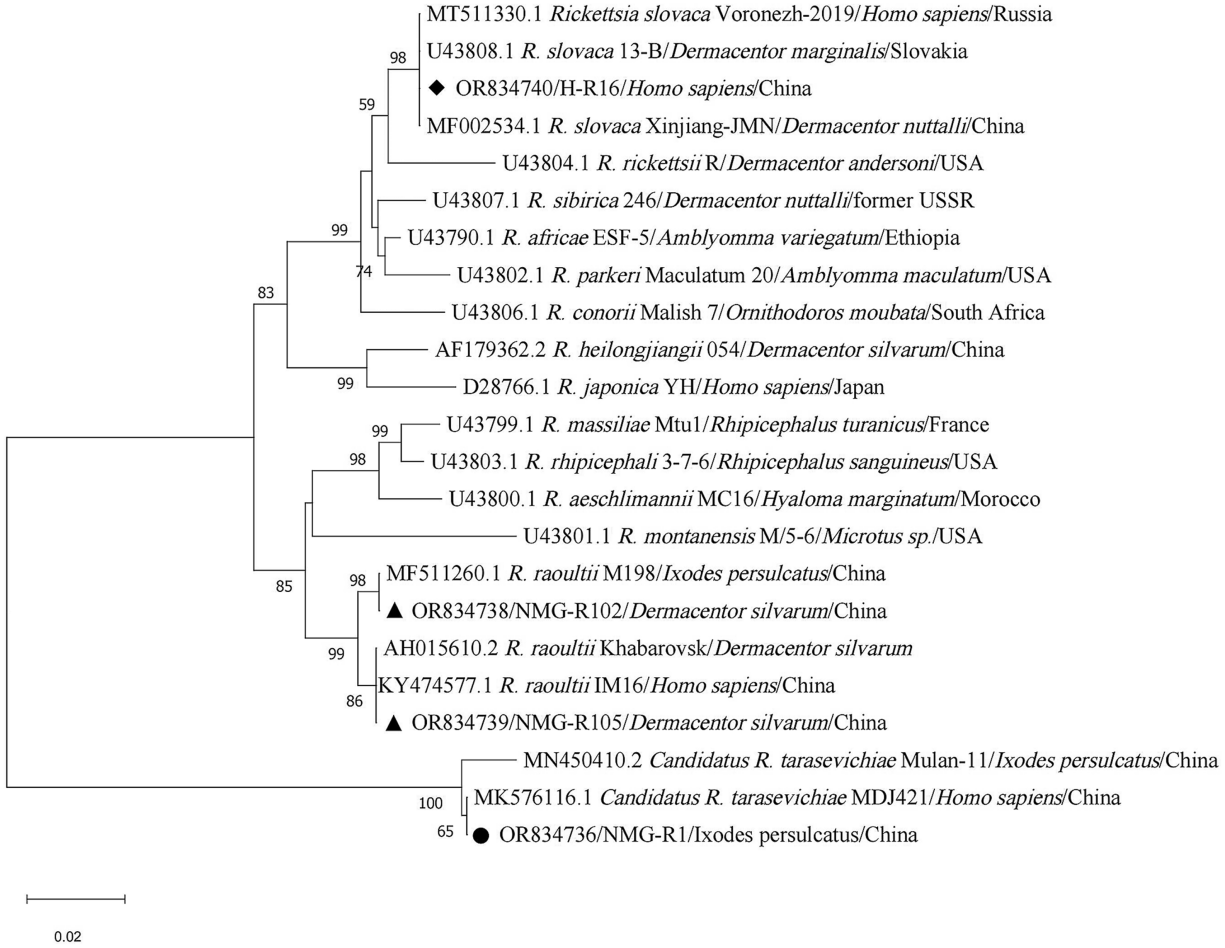
Phylogenetic analysis for *ompA* gene of SFGR. The sequences clustered as *Candidatus R. tarasevichiae* were marked as “●,” *R. raoultii* were marked as “▲,” *R. slovaca* were marked as “◆.”

**Figure 6:**
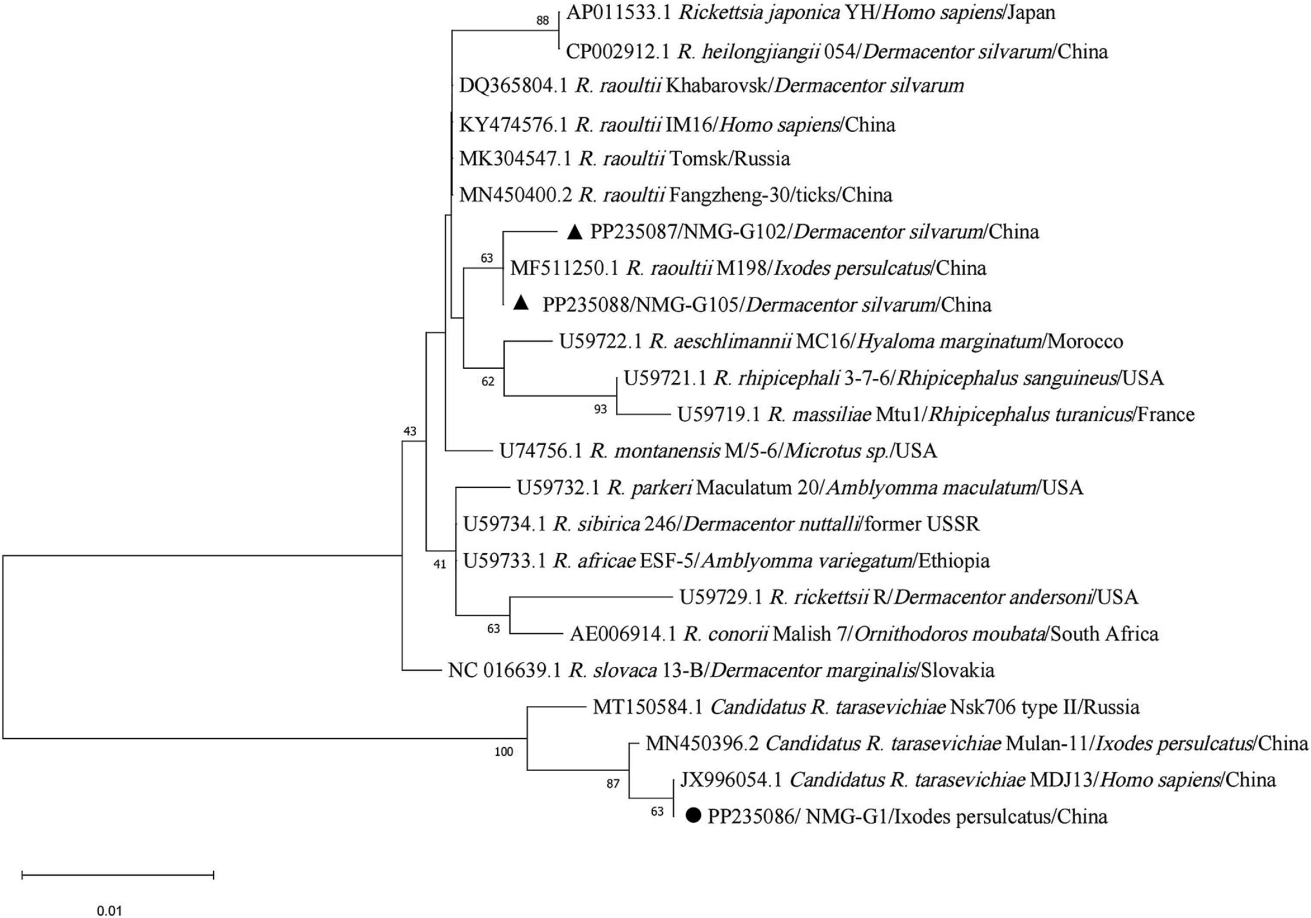
Phylogenetic analysis for *gltA* gene of SFGR. The sequences clustered as *Candidatus R. tarasevichiae* were marked as “●,” *R. raoultii* were marked as “▲.”

Phylogenetic tree based on *ompA* gene sequences of SFGR was shown in Figure 5. Of the 72 *I. persulcatus* positive for *Candidatus R. tarasevichiae*, all PCR amplicon sequences were identical (NMG-R1) and placed in a clade with *Candidatus R. tarasevichiae* from China (MN450410.2, MK576116.1). Two amplicon sequences (NMG-R102 and NMG-R105) detected in *D. silvarum* were closely related to strain Khabarovsk (AH015610.2, type strain of *R. raoultii*), and *R. raoultii* from China (KY474577.1 and MF511260.1). In particular, *R. slovaca* was detected in two individuals; both sequences were identical (H-R16) and clustered with strain 13-B (U43808.1, type strain of *R. slovaca*), and *R. slovaca* from China (MF002534.1), Russia (MT511330.1). In the phylogenetic tree based on *gltA* gene (Figure 6), 89 sequences amplified from *I. persulcatus* were identical (NMG-G1) and placed in a clade with *Candidatus R. tarasevichiae* from China (MN450396.2, JX996054.1). Two amplicon sequences (NMG-G102 and NMG-G105) detected in *D. silvarum* belonged to the same branch as *R. raoultii* from China (MF511250.1).

**Figure 7:**
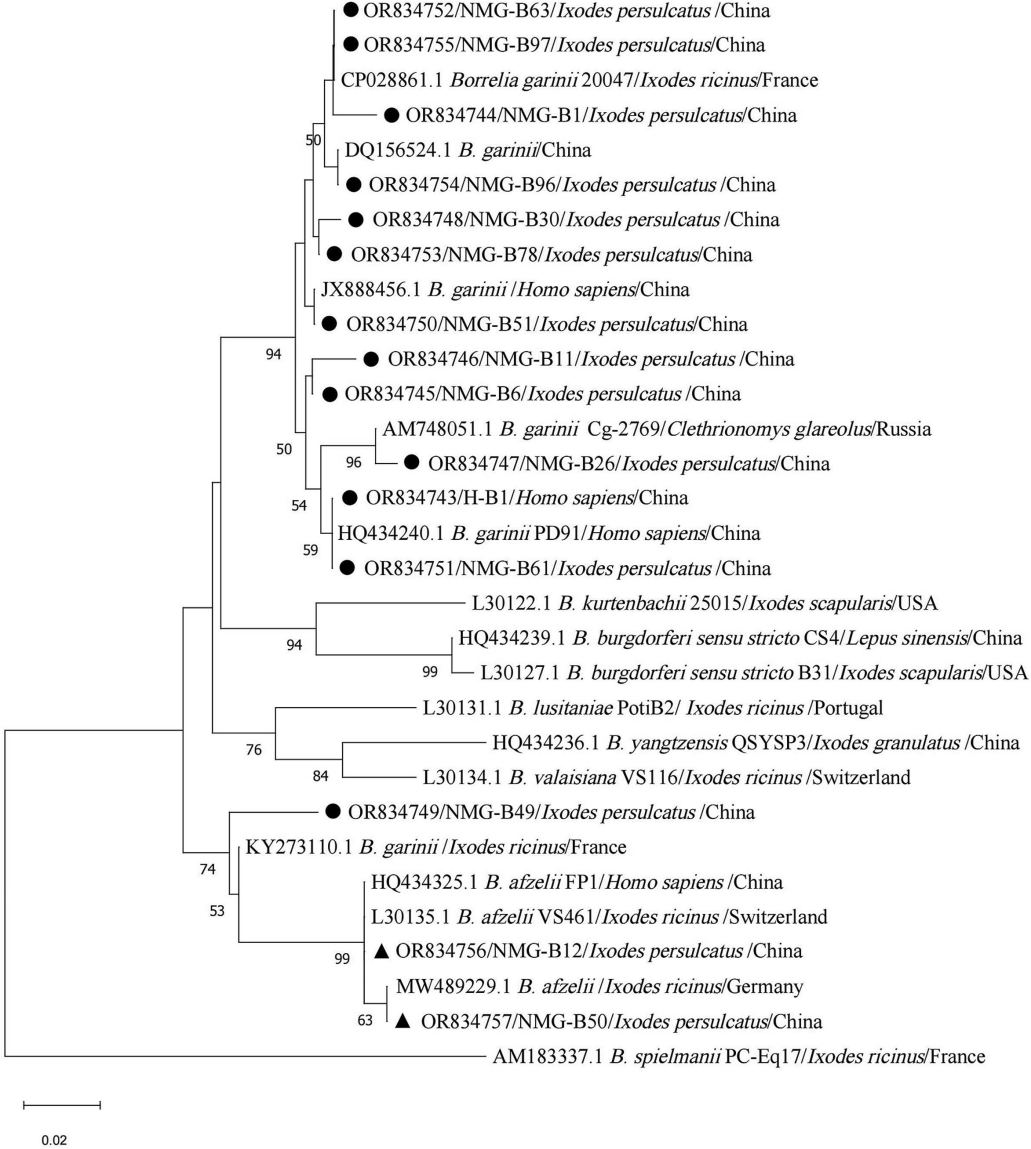
Phylogenetic analysis for 5S-23S rRNA intergenic spacer region of *B. burgdorferi sensu lato*. The sequences clustered as *B. garinii* were marked as “●,” *B. afzelii* were marked as “▲.”

Seventeen *I. persulcatus* were positive for *B. garinii*. After removing duplicate sequences, the remaining 12 sequences were used to build the phylogenetic tree of *B. burgdorferi*. The 11 sequences were found to be closely related to strain 20047 (CP028861.1, type strain of *B. garinii*), *B. garinii* from China (JX888456.1, DQ156524.1, and HQ434240.1), and Russia (AM748051.1), another sequence (NMG-B49) was clustered with *B. garinii* (KY273110.1) from France. Similarly, 12 human blood samples were identified as positive for *B. garinii*, and all sequences were identical to each other (H-B1) and matched 100% with the sequences (NMG-B61) from *I. persulcatus* and *B. garinii* strain PD91 (HQ434240.1). We detected *B. afzelii* in seven *I. persulcatus* samples; two sequences (NMG-B12 and NMG-B50) fell into the same branch as strain VS461 (L30135.1, type strain of *B. afzelii*), *B. afzelii* from China (HQ434325.1) and Germany (MW489229.1) after the elimination of equal sequences (Figure 7).

**Figure 8:**
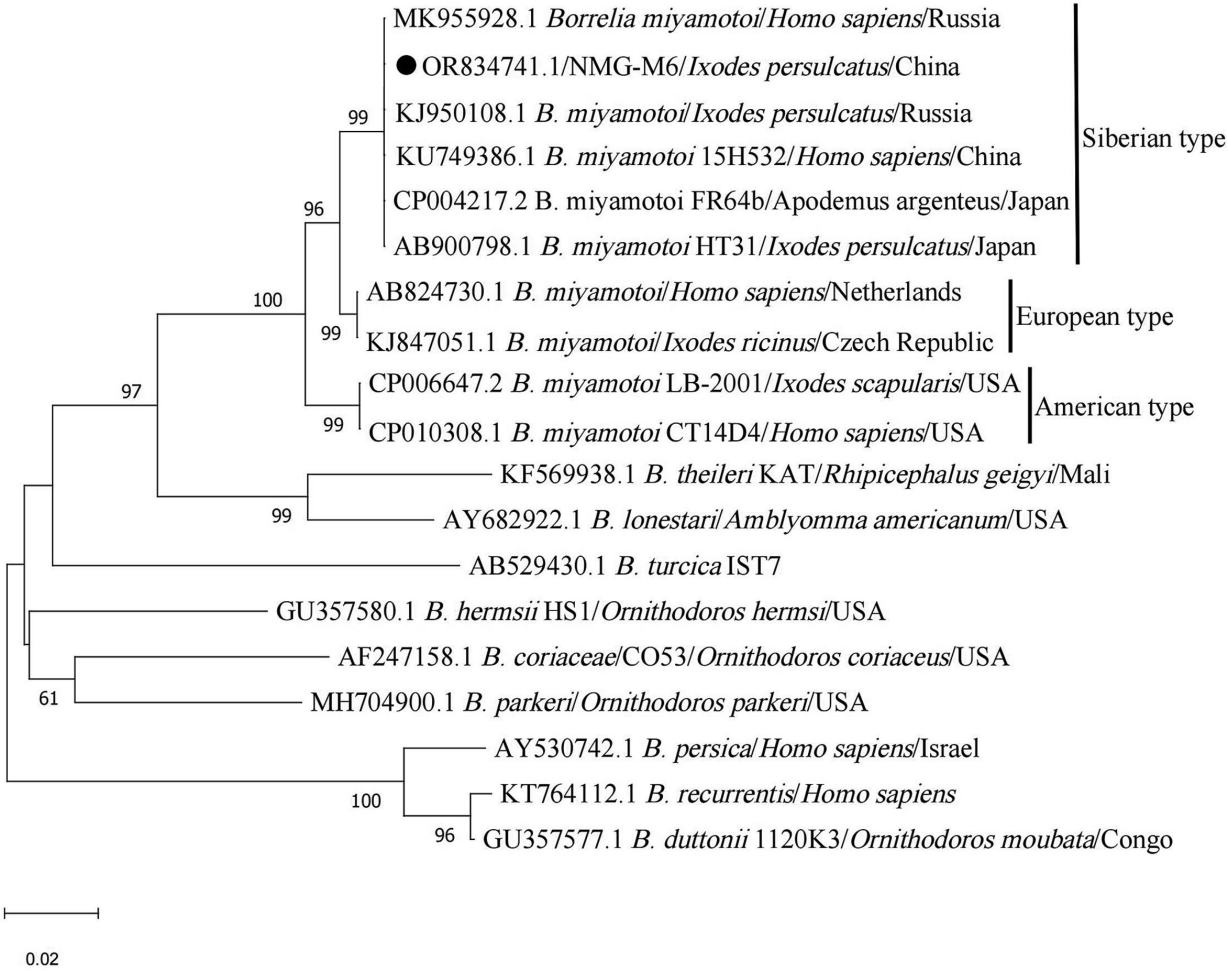
Phylogenetic analysis for *glpQ* gene of *B. miyamotoi*. The sequence clustered as *B. miyamotoi* was marked as “●.”

Among the seven *I. persulcatus* samples positive for *B. miyamotoi*, all sequences were identical (NMG-M6) and closely related to strain HT31 (AB900798.1, type strain of *B. miyamotoi*), *B. miyamotoi* from China (KU749386.1), Japan (CP004217.2), and Russia (MK955928.1, KJ950108.1), which belong to the Siberian type (Figure 8).

**Figure 9:**
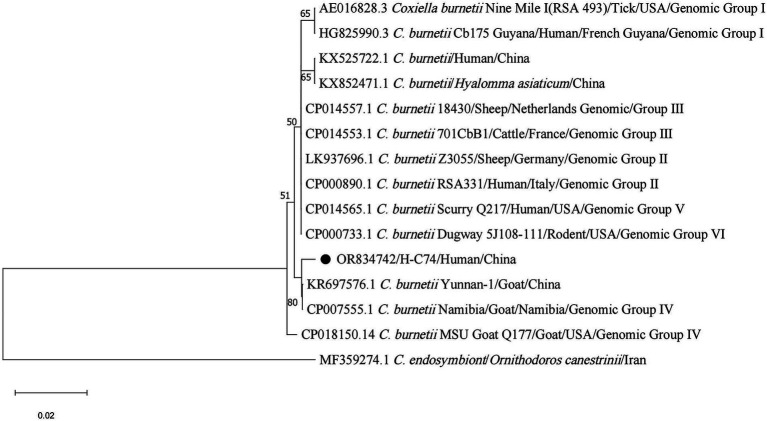
Phylogenetic analysis for *IS1111* gene of *C. burnetii*. The sequence clustered as *C. burnetii* was marked as “●.”

In addition, the sequence (H-C74) detected in a blood sample fell into the same branch as strain Namibia belonging to genomic group IV (CP007555.1), *C. burnetii* from China (KR697576.1) (Figure 9).

### Co-infection of pathogenic bacteria in ticks

In the present study, 27 *I. persulcatus* samples (27.00%, 27/100) were found to be co-infected with more than one bacterium (Table 3). Dual co-infection with *B. garinii* and *Candidatus R. tarasevichiae* (13.00%, 13/100) was most frequent in *I. persulcatus*. There was no co-infection in the participants according to PCR results.

**Table 3:** Prevalence of co-infection with tick-borne bacteria in *I. persulcatus*.

Bacteria	Number of ticks positive for co-infection (%)
Double	
B.garinii, Candidatus R. tarasevichiae	13 (13.00%)
B.afzelii, Candidatus R. tarasevichiae	7 (7.00%)
B.miyamotoi, Candidatus R. tarasevichiae	3 (3.00%)
Triple	
B.garinii, B.miyamotoi, Candidatus R. tarasevichiae	4 (4.00%)
Total	27 (27.00%)

### Seroprevalence of SFGR and *Borrelia burgdorferi*

Among the 245 serum samples, 14 were positive for anti-SFGR by ELISA, of which four were only positive for IgG and 10 were positive for IgM and IgG (Table 4). Both IgG and IgM antibodies against SFGR were negative in those 2 individuals who were detected positive for *R. slovaca* using PCR. Forty serum samples were seropositive for *B. burgdorferi* using IFA, and positive samples were further detected by WB; 33 of them were positive for anti-*B. burgdorferi* IgG (Table 4). The representative image of positive western blotting reactions was displayed in Additional file 5 (Supplementary Figure S1). Two serum samples were positive for both anti-*B. burgdorferi* IgG and anti-SFGR IgG.

**Table 4:** Seroprevalence of antibodies against *B. burgdorferi* and SFGR.

Antibody	Samples	*B. burgdorferi*		SFGR
		IFA	WB	ELISA
IgM	245	1 (0.41%)	–	–
IgG	245	38 (15.51%)	33 (13.47%)	4 (1.63%)
IgM and IgG	245	1 (0.41%)	–	10 (4.08%)
Total	245	40 (16.33%)	33 (13.47%)	14 (5.71%)

No significant difference in the seroprevalence of antibodies against SFGR and *B. burgdorferi* according to sex, age, or workplace was observed (Table 5).

**Table 5:** Differences in seroprevalence of antibodies against *B. burgdorferi* and SFGR according to sex, age, and workplace.

	No. tested	*B.burgdorferi*			SFGR		
		WB-Positive	Positive rate (%)	*p*	ELISA-Positive	Positive rate (%)	*p*
**Sex**							
Male	232	31	13.36	1.000	12	5.17	0.353
female	13	2	15.38		2	15.38	
Age							
21~	13	1	7.69	0.337*	–	–	0.202*
31~	27	3	11.11		4	14.81	
41~	51	11	21.57		2	3.92	
51~60	154	18	11.69		8	5.19	
**Workplace**							
Tianchi Forestry	21	3	14.29	0.628*	1	4.76	0.316*
Sandur Forestry	39	4	10.26		2	5.13	
Lixin Forestry	40	9	22.50		3	7.50	
Irsh Forestry	69	7	10.14		4	5.80	
Transportation Company	25	3	12.00		2	8.00	
Xing’an Service Area	22	4	18.18		4	18.18	
Jinjianggou Service Area	29	3	10.34		–	–	

*Fisher’s exact text.

## Discussion

Ticks are recognized as one of the most important vectors of a wide variety of diseases in humans and animals. In recent years, an increasing number of tick-borne infections have stimulated the investigation of ticks and tick-borne pathogens (31, 32). In this study, we applied 16S rDNA V3–V4 region high-throughput sequencing combined with species-specific PCR to detect tick-borne bacteria in free-living ticks and humans at high risk of tick-borne diseases in Arxan, Inner Mongolia, China. Subsequently, the seroprevalence of antibodies against SFGR and *B. burgdorferi* was determined to understand the infection rate of major tick-borne bacteria in the participants.

In this survey, the collected free-living ticks were mainly *I. persulcatus* with a small number of *D. silvarum*. Arxan City is located in northeastern China, which is mainly covered by coniferous and broad-leaved forests and is characterized by strong seasonality in temperature, providing a favorable habitat for *I. persulcatus*. The natural habitats of *D. silvarum* were characterized by middle to high elevations, shrub grasslands, and low precipitation during the driest month (2). In addition, we collected ticks in May 2020 and May 2021. The peak for *I. persulcatus* adults occurred in late May (33), while *D. silvarum* peaked in mid-April (34). Due to the limitation of the sampling season, sampling sites, and the number of ticks collected, only a small number of *D. silvarum* were collected.

High-throughput sequencing of 16S rDNA has been widely used to study microbial communities in ticks. For example, Runlsi et al. identified 189 genera in *Haemaphysalis longicornis* through high-throughput sequencing at the Illumina HiSeq platform, including *Anaplasma, Rickettsia*, and *Ehrlichia* (35). Jun et al. used high-throughput sequencing to study the microbial diversity of *D. nuttalli* and revealed the presence and relative abundance of the bacterial genera *Rickettsia*, *Anaplasma* and *Coxiella* (8). In the present study, at least 32 phyla, 79 classes, 191 orders, 348 families, and 594 genera were detected in pooled DNA samples of *I. persulcatus*, and the Ace and Chao1 indices were more than 1068.34 and 1098.66, respectively, in each pool indicating that all pools were of highly species richness. Simpson indices were more than 0.85 except for N23 indicating that almost all pools were highly diverse. Of 594 genera, *Rickettsia*, *Anaplasma*, *Coxiella*, and *Borrelia*, were detected in pooled DNA samples of *I. persulcatus* through high-throughput sequencing of the 16S rDNA V3–V4 region, *Rickettsia* was recognized as the most common genus. *Candidatus R. tarasevichiae* and three *Borrelia* species (*B. garinii*, *B. afzelii* and *B. miyamotoi*) were identified in *I. persulcatus* by nested PCR, whereas only *R. raoultii* was detected in *D. silvarum*.

*Candidatus R. tarasevichiae* was first detected in *I. persulcatus* collected from the southern Urals and Siberia in 2003 (36) and was then found in *Haemaphysalis japonica* and *D. silvarum* from the Russian Far East (37, 38). Human cases of *Candidatus R. tarasevichiae* infection were first identified using laboratory molecular testing in eastern China in 2012 (39). In the present study, *Candidatus R. tarasevichiae* was detected in 89.00% of *I. persulcatus*, which was considered the dominant genotype of SFGR carried by *I. persulcatus* in Arxan.

*Rickettsia solvaca* was first isolated from *D. marginatus* ticks in Slovakia in 1968, and subsequent investigations reported that *R. solvaca* is widely distributed in *Dermacentor* ticks (40). Tian et al. firstly detected *R. slovaca* in *D. silvarum* collected from Xinjiang Autonomous Region, China in 2012 (41). In the present study, *R. slovaca* was detected in two participants using nested PCR targeting *ompA* gene. This is the first study to report the presence of *R. slovaca* has been found in humans in Inner Mongolia, China.

*Rickettsia raoultii* was first detected in *Dermacentor* ticks collected in Russia in 1999 (42) and then isolated from *Dermacentor* ticks in France, and named in 2008 (43). Similar to *R. slovaca*, *R. raoultii* was more common among *Dermacentor* ticks. Other hard ticks infected with *R. raoultii* have been reported by several groups in recent years, including the *Haemaphysalis*, *Hyalomma*, and *Ixodes* ticks (44–46). In this study, *R. raoultii* was detected in nine *D. silvarum* using nested PCR.

*Candidatus R. tarasevichiae*, *R. slovaca*, and *R. raoultii* were all pathogenic genotypes of spotted fever group Rickettsia. The SFG patients present with rashes, eschar, fever, fatigue, anorexia, nausea, and local lymph node enlargement. A few patients had neurological manifestations such as coma, neck stiffness, and Kernig’s sign (47). Combined with the presence of anti-SFGR antibodies among participants, public health workers in the area need to be aware of the risk of SFGR infection; regularly conduct education campaigns on tick bite prevention; and monitor populations, ticks, and animal hosts.

*Borrelia burgdorferi*, first isolated from *I. persulcatus* in 1982 (48), was the causative pathogen of Lyme disease. In our previous study, two *B. garinii* strains were isolated from ticks in Arxan, suggesting that this area was the natural focus of Lyme disease (49). Through nested PCR targeting 5S-23S rRNA IGS, *B. garinii* was the dominant genotype of *B. burgdorferi* carried by *I. persulcatus* collected from Arxan, followed by *B. afzelii*, and the two genotypes were dominant causative genotypes of *B. burgdorferi* in China. We also detected *B. garinii* in 12 participants, and these 12 sequences were identical to the sequence (NMG-B61) detected in *I. persulcatus*, indicating the transmission of *B. garinii* from ticks to humans in Arxan. The positivity rate of anti-*B. burgdorferi* in participants was 13.47% based on the IFA results and confirmed by Western Blot assay, which was similar to the positive rate of forestry populations in Inner Mongolia (15). Human Lyme disease generally occurs in stages, from the early localized stage of erythema migrans, fatigue, chills, and fever, to a late disseminated stage of intermittent bouts of arthritis with severe joint pain and swelling and neurological symptoms (29). Thus, Lyme disease should be considered by clinicians with the forestry workers who had correlated symptoms with Lyme disease in Arxan.

*B. miyamotoi*, initially identified and isolated in Japan in 1994 (50), is the causative agent of *B. miyamotoi* disease with generalized flu-like symptoms. Human cases of *B. miyamotoi* infection have been reported in European countries (51, 52), the United States (53, 54), Australia (55), and Japan (56). A survey of *B. miyamotoi* in Inner Mongolia suggested that 2.6% of *I. persulcatus* carried *B. miyamotoi* and 1.7% of patients bitten by ticks were infected with *B. miyamotoi* (57). In this study, *B. miyamotoi* which belongs to the Siberian type, was detected in 7% of *I. persulcatus*. This is the first report of this bacterium that has been detected in ticks from Arxan, indicating that local forestry populations are at risk of *B. miyamotoi* infection.

*Coxiella burnetii*, the causative agent of Q fever, is transmitted to humans via inhalation of infected aerosols or tick bites (58). In our study, *C. burnetii* was detected in one participant, indicating that extensive and in-depth monitoring of *C. burnetii* in ticks and animal hosts should be conducted to identify the risk of infection in the local population.

Ticks can acquire multiple pathogenic species during blood feeding on their vertebrate hosts, and humans may be infected by more than one pathogen carried by ticks. In this study, *Candidatus R. tarasevichiae* and *B. garinii* were the dominant pathogens detected in *I. persulcatus*, which, to some extent, explains why the rate of co-infection with *B. burgdorferi* and SFGR was the highest in *I. persulcatus*. Two participants tested positive for antibodies against both *B. burgdorferi* and SFGR, indicating that cases of coinfection with *B. burgdorferi* and SFGR were present in Arxan, Inner Mongolia. In recent years, concurrent infections with multiple tick-borne agents have been reported, including co-infection with *B. burgdorferi s.l* and *A. phagocytophilum* (59), *Babesia microti* and *B. burgdorferi s.l* (60), SFGR and severe fever with thrombocytopenia syndrome virus (61), etc. Pathogens may behave synergistically, indifferently, or antagonistically within common human hosts, thus prolonging the duration of symptoms and modulating disease severity (31). Additionally, the presence of concurrent infections may be neglected when multiple pathogens are encountered, which results in missed diagnoses and poses an additional challenge for a definitive diagnosis. Therefore, when treating patients with suspected tick-borne diseases, clinicians in this area should consider the possibility of coinfection. Long-term monitoring of tick-borne pathogens in ticks and humans should be investigated in the future.

## Data availability statement

The datasets presented in this study can be found in online repositories. The names of the repository/repositories and accession number(s) can be found at: [https://www.ncbi.nlm.nih.gov/ AND OR841358–OR841362 for *I. persulcatus*, OR841363–OR841367 for *D. silvarum*].

## Ethics statement

The studies involving humans were approved by the ethics committee of National Institute for Communicable Disease Control and Prevention, Chinese Center for Disease Control and Prevention, Beijing, China. The studies were conducted in accordance with the local legislation and institutional requirements. The participants provided their written informed consent to participate in this study. The manuscript presents research on animals that do not require ethical approval for their study.

## Author contributions

LD: Data curation, Formal analysis, Validation, Visualization, Writing – original draft. LZ: Investigation, Formal analysis. XH: Investigation, Methodology. ZB: Methodology. YZ: Investigation. LH: Methodology. ZL: Methodology. HZ: Supervision, Writing – review & editing. QH: Project administration, Supervision, Investigation, Formal analysis, Writing – original draft, Writing – review & editing. AD: Supervision, Writing – review & editing.

## Footnotes

^1^ https://blast.ncbi.nlm.nih.gov/Blast.cgi
