# Supplementary figures and images for: Surveillance of tick-borne bacteria infection in ticks and forestry populations in Inner Mongolia, China
Source: Front Public Health. 2024 Feb 29;12:1302133. doi: 10.3389/fpubh.2024.1302133 (PMC10938913; doi:10.3389/fpubh.2024.1302133)

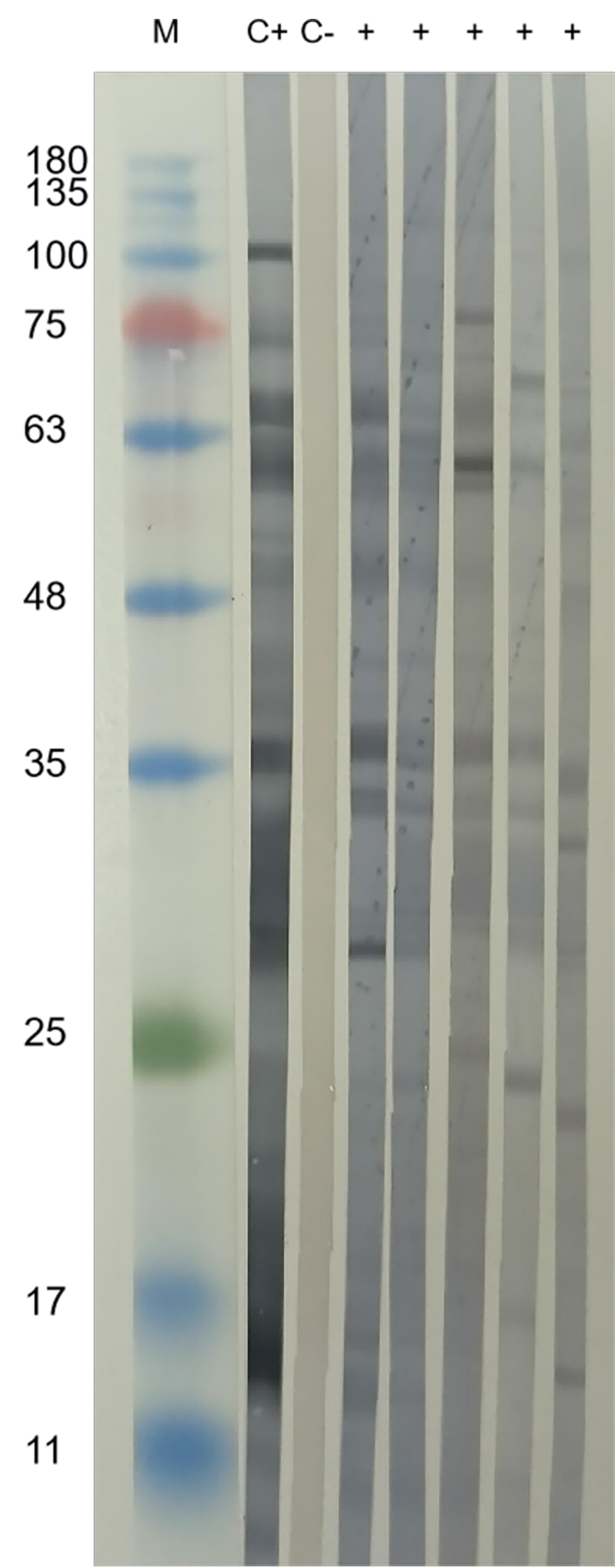

Supplement: Supplementary file 5 [file Image_1.tif]
